# Functionalized Superparamagnetic Iron Oxide Nanoparticles (SPIONs) as Platform for the Targeted Multimodal Tumor Therapy

**DOI:** 10.3389/fonc.2019.00059

**Published:** 2019-02-13

**Authors:** Christina Janko, Teresa Ratschker, Khanh Nguyen, Lisa Zschiesche, Rainer Tietze, Stefan Lyer, Christoph Alexiou

**Affiliations:** ^1^Department of Otorhinolaryngology, Head and Neck Surgery, Section of Experimental Oncology and Nanomedicine (SEON), Else Kröner-Fresenius-Stiftung Professorship, Universitätsklinikum Erlangen, Erlangen, Germany; ^2^Friedrich-Alexander-Universität Erlangen-Nürnberg (FAU), Erlangen, Germany

**Keywords:** nanoparticles, nanomedicine, targeted therapy, immunotherapy, chemotherapy, irradiation, immunogenic cell death

## Abstract

Standard cancer treatments involve surgery, radiotherapy, chemotherapy, and immunotherapy. In clinical practice, the respective drugs are applied orally or intravenously leading to their systemic circulation in the whole organism. For chemotherapeutics or immune modulatory agents, severe side effects such as immune depression or autoimmunity can occur. At the same time the intratumoral drug doses are often too low for effective cancer therapy. Since monotherapies frequently cannot cure cancer, due to their synergistic effects multimodal therapy concepts are applied to enhance treatment efficacy. The targeted delivery of drugs to the tumor by employment of functionalized nanoparticles might be a promising solution to overcome these challenges. For multimodal therapy concepts and individualized patient care nanoparticle platforms can be functionalized with compounds from various therapeutic classes (e.g. radiosensitizers, phototoxic drugs, chemotherapeutics, immune modulators). Superparamagnetic iron oxide nanoparticles (SPIONs) as drug transporters can add further functionalities, such as guidance or heating by external magnetic fields (Magnetic Drug Targeting or Magnetic Hyperthermia), and imaging-controlled therapy (Magnetic Resonance Imaging).

## Evolvement of Tumors and Their Treatments

Mutation and clonal selection are driving forces in carcinogenesis (1). Accumulation of mutations in proto-oncogenes and tumor suppressor genes lead to uncontrolled proliferation of cells. Some of these mutations are recognized by the immune system as “non-self” (tumor associated antigens) and are eliminated, a process known as “immunosurveillance” (2, 3). Cells expressing only low amounts of tumor associated antigens cannot be detected and removed. Thus, the immune system exerts a selective force on the tumor, altering cell composition and promoting survival of the least immunogenic cells (“immunoediting”) (4). Tumors evade the immune system by various mechanisms such as downregulation of MHC I expression, development of resistance to cytotoxic T lymphocytes, active suppression of activated T cells, or release of immune suppressive molecules (5). In the clinic, tumors are treated by surgery, radiotherapy, chemotherapy, photodynamic therapy, and others. All of these procedures can induce the release of immune stimulatory intracellular molecules increasing the immunogenicity of the tumor. Immunotherapies shall further intensify the strength of immune responses. Problematically, monotherapies often cannot remove the tumor completely due to the occurrence of resistant tumor cell populations. Chemotherapy can lead to multiple drug resistance in long term use (6). In radiotherapy the lack of oxygen in hypoxic tumor tissues results in reduced production of reactive oxygen species (ROS) and thus decreased DNA damage (7). Immunotherapy is often effective only in a subgroup of patients. Thus, combinations of therapy concepts exhibiting synergistic effects might overcome limitations of monotherapies, referred to as multimodal tumor therapy. To bring therapeutics to the tumor area, nanoparticles have come into focus. Serving as transporters, various therapeutic cargos can be integrated in one nanoparticle system to combine different functionalities. Here we discuss the use of nanoparticles as multimodal drug transporters with special emphasis on superparamagnetic iron oxide nanoparticles (SPIONs). Based on their magnetic core they can be magnetically guided to the desired place, visualized in magnetic resonance imaging (MRI) and serve as heat transporters in magnetic hyperthermia.

## Challenges of Systemic Tumor Therapies

After intravenous or oral application of fluid chemo- and immunotherapeutics, the drug circulates in the whole organism and only a fraction reaches the tumor, whereas the majority disappears in the healthy tissues or is ejected. Thus, high doses must be applied for sufficient therapeutic concentrations in the tumor (8). Also, poor solubility can be an obstacle to reach effective therapeutic doses.

Chemotherapeutics are injected in a cyclic schedule to kill the rapidly proliferating tumor cells. Problematically, not only the tumor is affected but also healthy tissues (9) with quickly dividing cells such as cells of the blood, the immune system, hair, or mucosa. Since some cytostatic agents are carcinogens themselves they sometimes induce acute myeloid leukemia after therapy (9). Additionally, the risk of chemotherapy-associated anemia (10) and neutropenia (11) is high. Thus, immune function must be monitored regularly. In case of severe limitations, it may be necessary to reduce or stop the therapy (12). If the number of leukocytes in blood is too low, infections may occur and therefore patients often die due to therapy-related side effects and not the tumor itself (13).

Unlike chemotherapy, immunotherapy does not destroy cancer cells directly. The goal of immunotherapy is to manipulate the immune system to kill cancer without impeding normal tissues. Since checkpoint inhibitors act by blocking the inhibition of T cells, additionally to the wanted reactions such as tumor infiltration and killing of cancer cells, activated T cells can also attack healthy cells, resembling autoimmune reactions (14). While chemotherapy is associated with immunosuppression and infections, some of the recent approaches in immunotherapy can be accompanied by massive inflammatory responses and autoimmune-type like pathologies, which can affect all the organs of the body (14, 15). For Ipilimumab therapy in metastatic melanoma for instance, immune-mediated side effects as dermatitis, hepatitis, enterocolitis, hypophysitis, and uveitis, which can be life threatening, have been described (16). For management of inflammatory side effects systemic steroids or corticosteroids should be considered (16).

Immunotherapies are effective only in a subgroup of cancers and a minority of patients (17, 18). Reasons for this are tumor heterogeneity, previous treatments, variability in tumor type and stage and immunosuppressive phenotype of the cancer (19). Tumors with many mutations seem to have better response rates to immune checkpoint blockade with PD-1, probably due to higher tumor immunogenicity (20). Since immunotherapies are not applied as first line treatments, they are rather given to patients with compromised immune systems due to advanced disease or previous chemotherapy cycles, hindering the development of effective immune reactions (21).

Moreover, immunotherapies are very expensive depending on dosing and scheduling, putting economic pressure on patient and healthcare system (22). In 2016 the one-year per-patient costs for treatment of metastatic melanoma with PD-1 inhibitor Pembrozulimab was $145,010, achieving a progressing-free survival of 6.3 month (23, 24). Combination therapies can even double or triple the costs. These extremely expensive therapies might be denied by health insurances or lead to restrictions for patients who cannot afford additional payments for the drugs (24). Also, only few of the treatments reach complete tumor remission after one treatment cycle, so that multiple rounds of treatments are necessary.

## Targeted Therapies Using Nanoparticles

Systemic toxicities can hinder the efficacy of potent antitumor drugs. However, side effects caused by the unspecific distribution and low doses in the target area are not only problems in the treatment of tumors but also of various other diseases. To bring therapeutics directly to the target area and to reduce systemic concentrations nanocarriers have come into focus.

### Passive Delivery of Nanoparticles

Distribution, pharmacokinetics and retention of medical nanoparticles strongly depend on the route of application and the physicochemical nanoparticle characteristics. For daily medication, oral application is comfortable for the patients. However, orally applied nanoparticles are rather quickly excreted from the body than being absorbed through the intestine into the blood. A possibility to increase nanoparticle absorption from the gastrointestinal tract is the conjugation of nanoparticles with bile acids, employing bile acid transporter-mediated cellular uptake and chylomicron transport pathways (25). With intravenous application nanoparticles tend to be restricted to the vascular system and to organs with a fenestrated endothelium, such as liver and spleen since the pore size of normal intact endothelium is about 5 nm. Tumors and inflamed areas are accessible as well, since they exhibit fenestrated endothelium and vascular leakiness. Depending on their size, injected nanoparticles undergo renal clearance including glomerular filtration, tubular secretion, and finally elimination through urinary excretion. For globular proteins the filtration-size threshold is < 5 nm, and this seems to be comparable for nanoparticles (26, 27). Larger particles are cleared from blood circulation via phagocytic cells of the reticuloendothelial system (RES). Macrophages in the liver (Kupffer cells), the spleen and the circulating blood rapidly take up opsonized nanoparticles and intracellularly degrade them (28, 29). Importantly, systemic inflammation affects nanoparticle distribution by alteration of systemic circulatory properties, modulation of the immune system and increase of vessel permeability (30). Modification of the nanoparticle surface by polyethylene glycol (PEG) reduces non-specific protein adsorption and opsonization and minimizes clearance by the RES, thus resulting in longer blood circulation times and improved pharmacokinetic properties (31). Intraarterial injection in proximity to the tumor site can limit the nanoparticle removal by the RES (32).

When tumors exceed a distinct size transport of oxygen and nutrients by diffusion is insufficient and access to the blood circulation is necessary (33). Contrary to healthy blood vessels, tumor capillaries have large gaps between endothelial cells, a wide irregular lumen and lack of smooth muscle cells, enabling the selective extravasation. The poor lymphatic drainage permits retention of macromolecular drugs or nanoparticles in the tumor microenvironment, referred to as enhanced permeation and retention (EPR) effect (34, 35). So far, several clinically approved chemotherapeutics such as doxorubicin, daunorubicin, or vincristine encapsulated into liposomes have been approved as nanomedicines by the Federal Drug Administration (FDA). Beside these first generation clinically approved nanomedicines, other non-targeted nanosystems are under investigations in clinical studies (phase I/II/III) (36).

### Active Delivery of Nanoparticles

Despite preferential accumulation in tumor tissues due to the EPR effect, the fraction of nanoparticles finally entering the tumor is still limited. The majority of the applied nanoparticles is removed from blood in a few hours and only some percent remain in the systemic circulation (37). Finally, only ~2% of the total intravenously administered dose is deposited in the tumor after 4 h of circulation (38). To increase the intratumoral dose, several studies revealed receptor-based active targeting of nanoparticles to be a promising delivery strategy (39). Targeting ligands such as monoclonal antibodies and antibody fragments, aptamers, peptides and small molecules are under extensive investigation for use in diagnostics, therapy and post-therapeutic follow-up (40). For example, Trastuzumab functionalized nanoparticles targeting Her2 positive tumor cells showed favorable results in experiments with breast cancer cells as diagnostic agents and drug delivery vehicles (41, 42). SPIONs with folic acid as targeting molecule enhanced the uptake by folate receptor exposing tumor cells (43).

Beside use of targeting moieties, nanoparticles can be transported by physical forces to the desired place. For instance, SPIONs can be applied as drug transporters in Magnetic Drug Targeting (MDT). To prevent clearance by RES, SPIONs are applied intraarterially in the tumor supplying vascular system and are enriched in the tumor region using an external magnetic field. Previously, studies with tumor bearing rabbits (squamous cell carcinoma) revealed that the amount of the chemotherapeutic agent mitoxantrone in the tumor region can be increased from 1% after intravenous application to 50–60% with MDT. Complete tumor remissions or slower tumor growth with increased survival times were shown in the majority of the treated animals (44). Also, immune cells from peripheral blood were spared from the toxic effects of the chemotherapy, due to specific accumulation in the tumor (45).

A major challenge remains the treatment of tumors in the brain, due to often being surrounded by important functional structures, which can be injured by interventions such as surgery, intratumoral injections or radiation. In this case, magnetic forces can be used to trap SPIONs at the site of interest. To bypass the first-pass organ clearance of the magnetic nanoparticles, intraarterial administration via carotid artery enhances nanoparticle exposure of the tumor vasculature. Together with an MRI guided subject alignment within the magnetic field and surface modification of the drug with biological membrane permeable polyethyleneimine it is possible to deliver ß-galactosidase selectively to the brain tumor in a rat glioma model, while limiting the exposure of healthy brain areas (32). In this approach, magnetic field topography is essential to prevent magnetic aggregation in the vasculature (32, 46, 47). To prevent nanoparticle aggregation and occlusion of vessels in magnetic fields we found that a proper surface coating and colloidal stabilization of SPIONs is a prerequisite (48).

## Immunogenic Cell Death Induction by Tumor Therapies

**Figure 1:**
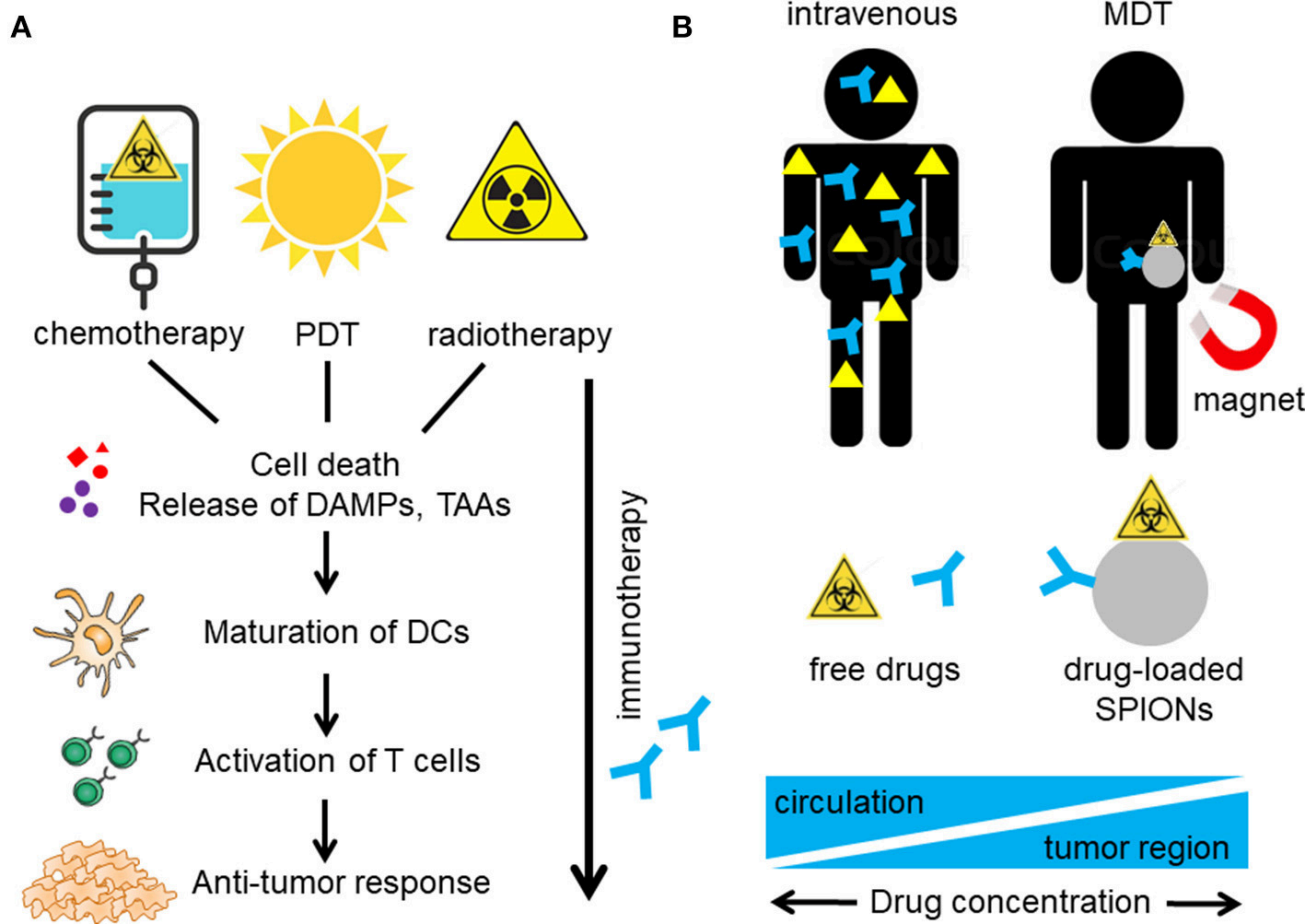
Induction of anti-tumor immune reactions by multimodal therapy. **(A)** Chemotherapy, radiotherapy, and photodynamic therapy (PDT) induce immunogenic cell death (ICD) in the tumor with release of damage associated molecular patterns (DAMPs) and tumor associated antigens (TAA). TAA are taken up by antigen presenting cells (APC), such as dendritic cells (DCs) and are processed and presented to T cells, which are activated to proliferate. Accompanying immunotherapy (e.g., with anti-PD-1) blocks PD-1 (on T cells) and PD-L1 (on tumor cells and APCs) interaction, resulting in immune activation and increase of anti-tumor immune responses. **(B)** Integrating several treatment functionalities on one nanoparticle and active targeting to the tumor region e.g. by magnetic drug targeting (MDT) might increase the therapeutic doses in the tumor and reduce systemic distribution with accompanying side effects such as immune deprivation.

Therapeutic strategy of conventional treatments relies on the rationale that rapidly proliferating tumor cells are more sensitive to toxic chemicals or radiation than healthy tissues. In the past, it has been believed that these treatments simply act by killing the tumor cells or inhibiting their proliferation. However, it became apparent that distinct cell death pathways activated during cell stress turn the cells “visible” for the immune system, a process referred to as immunogenic cell death (ICD). Agents inducing ICD in cancer therapy are for example chemotherapeutics from the class of the anthracyclines and their derivatives (e.g., doxorubicin, mitoxantrone), photosensibilisators for photodynamic therapy (PDT) or radiotherapy (49). In contrast to apoptosis, the physiological form of cell death, eliciting inflammatory silent or even anti-inflammatory clearance, ICD induces inflammatory immune reactions. Hallmark of ICD is the release of damage associated molecular patterns (DAMPs) from the dying cells in a timely resolved fashion (50). The early cell surface exposition of calreticulin, the active release of heat shock proteins (HSPs) and ATP as well as the *post mortem* leakage of HMGB1 have been described to act as endogenous adjuvants, recruiting and activating immune cells. Professional antigen presenting cells take up tumor derived antigens, process them, migrate to the tumor draining lymph nodes and cross present them to T cells. Subsequently, antigen specific T cells differentiate to effector T cells, proliferate, and are attracted to the tumor region by chemokines (51). There, effector T cells kill the tumor cells via cytotoxic granules or Fas-induced apoptosis and thereby create a new wave of released tumor antigens which boost the immune response (52). By inducing ICD radiation, photodynamic therapy (PDT) and/or chemotherapy may activate immune responses and immunize a patient against cancer by turning the tumor into an *in situ* vaccine (53). Radiation and chemotherapy both can induce DNA damage resulting in cell cycle arrest and/or cell death. Furthermore, cellular mutations with the development of neoantigens are provoked, resulting in higher immunogenicity (Figure 1A).

## Nanoparticle-Based Therapies

**Figure 2:**
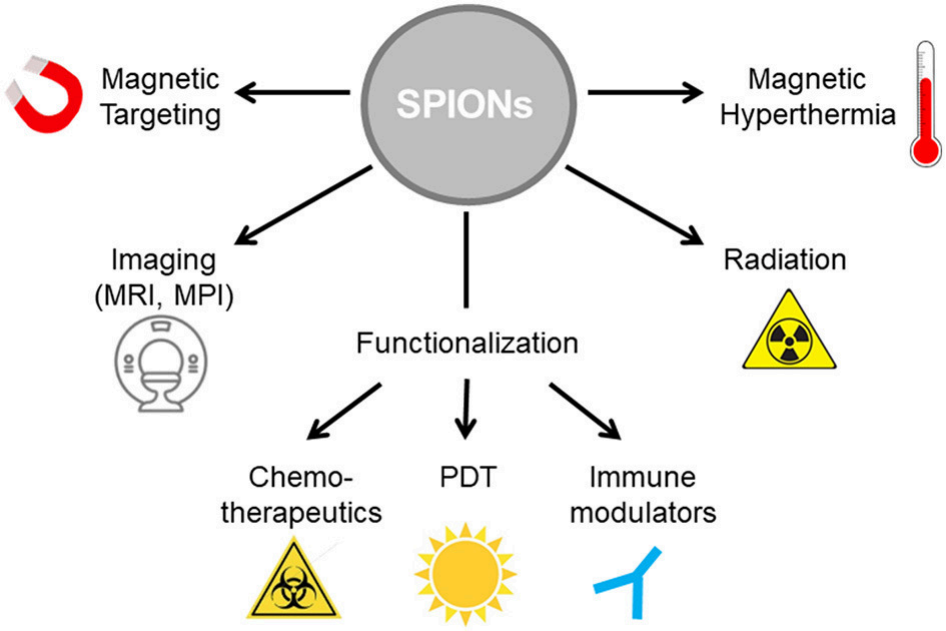
SPIONs as nanoparticle platform for multimodal tumor therapy. SPIONs can be functionalized with various cargos such as cytotoxic agents for chemotherapy, photosensibilisators for photodynamic therapy and/or immune modulators for immunotherapy. To increase treatment efficacy, magnetic hyperthermia can be induced in alternating magnetic fields. Radiation induces release of ROS on the particle surface. Imaging controlled therapy is enabled by magnetic resonance imaging (MRI).

Due to induction of ICD by several routine treatment regimens, the combination of those therapies with immunotherapeutic agents can induce or increase anti-tumor responses from the immune system. A multitude of various nanoparticle systems has been developed for medical application and multimodal tumor therapy, which are discussed elsewhere (54). SPIONs can be tailored in size, morphology and functionalization, enabling their use in a wide range of applications (55). SPIONs can be loaded as drug transporters with various cargos (chemotherapeutics, photosensibilisators, immune modulators), serve as contrast agents in MRI, provide heating capacity in alternating magnetic fields, and enable magnetic targeting (Figure 2). Due to these additional possibilities, a special focus will be set on SPIONs here.

### SPIONs as Drug Transporters

Prerequisite for use of nanoparticles in biomedicine is their biocompatibility. Due to their inorganic nature, SPIONs on their own are not sufficiently biocompatible. One strategy to circumvent this compatibility issue is to coat the SPIONs with biocompatible polymers (56). For SPIONs comprehensive studies have been performed with partially contradictory results dependent on size, coating, applied concentration and exposure time of the nanoparticles (57). Reported toxicities in experimental studies include reduced mitochondrial activity, cellular stress mediated generation of ROS, inflammation and chromosome condensation (58). In our hands, coating of nanoparticles with biocompatible substances such as crosslinked dextran or formation of an artificial protein corona of serum albumin not only increased colloidal stability of the particles but also their biocompatibility (59–64). Some formulations of magnetite-based nanoparticles have already been approved for use in humans as iron deficiency therapeutics and as MRI contrast agents by the FDA (e.g., Feraheme®, Feridex I.V.® and Gastromark®) (65). Once the SPIONs are administered intravenously, they enter liver and spleen (66). SPIONs are taken up into the lysosmes of cells, where the iron oxide is broken into iron ions presumably due to hydrolysing enzymes effective at low pH and ultimately get incorporated into hemoglobin (57, 67).

### Combination of Nanocarriers (SPIONs) With Chemotherapy

Challenges in routine chemotherapy are systemic toxicities. Despite several chemotherapeutics have shown the ability to induce ICD, systemic applications are accompanied by severe side effects, in particular destruction of the immune system (11). That's why some of the current chemotherapeutics are also used as immunosuppressive agents (e.g., cyclophaosphamide, methotrexate) for the treatment of severe autoimmune diseases. By loading chemotherapeutic drugs onto nanoparticles this challenge can be addressed. With targeting of nanoparticles to the tumor region, the systemic concentration is reduced while effective intratumoral doses are increased. Several chemotherapeutic agents such as doxorubicin, daunorubicin, or vincristine encapsulated into (PEGylated) liposomes have been approved as e.g., Doxil®/Caelyx®, DaunoXome®, or Marqibo, respectively. Paclitaxel bound to lyophilized human albumin as carrier protein is registered as Abraxane® for breast cancer treatment (68). Presensitization of tumor cells with antisense miRNA (against miRNAs expressed during cancer) or siRNA (against a developmental transcription factor reactivated in cancers) prior to chemotherapy can reduce the effective doses of chemotherapeutics needed or can overcome chemoresistance (69, 70).

To induce anti-tumor immune reactions, inducers of ICD such as oxaliplatin or doxorubicin have been loaded into nanocarriers (71–74). Exemplarily, after intravenous injection of oxaliplatin or doxorubicin-loaded amphiphilic diblock copolymer nanoparticles, the nanoparticle-encapsulated ICD inducer led to significantly enhanced ICD and consequently improved anti-tumor effects in pancreatic cancer xenograft compared to the free form (71). Active targeting of nanoformulations using magnetic forces have been explored to maximize drug accumulation of ICD inducers as well. We and others loaded chemotherapeutic drugs such as mitoxantrone or doxorubicin onto SPIONs and showed improved targeting and anti-tumor efficacy in the presence of magnetic fields *in vivo* (44, 75, 76). When we treated rabbits suffering from induced squamous cell carcinomas with SPIONs functionalized with mitoxantrone and targeted the particles to the tumor by an external magnet, the tumors were continuously shrinking until complete tumor disappearance after several weeks, indicating rather an immunological process than immediate tumor lysis by mitoxantrone (44). We proved that mitoxantrone functionalized SPIONs can induce ICD with concomitant release of DAMPs such as HSPs, ATP, HMGB1, and foster maturation of DCs (77).

Improving chemotherapy (probably by synergistically inducing ICD), pH sensitive magnetically guidable iron oxide nanocarriers loaded with doxorubicin and a photosensibilisator showed beneficial effects in U87 tumor bearing nude mice, thus overcoming chemoresistance (78).

### Combination of Nanocarriers (SPIONs) With Immunotherapy

Anti-cancer immunotherapies shall increase the strength of immune responses against the tumor by either stimulating activities of the immune system or block signals produced by cancer cells to suppress immune responses. In the evolving field of immunotherapy, therapeutic antibodies against tumor antigens (e.g., Herceptin targeting HER-2/neu on breast cancer) or antibodies inhibiting the proliferation of tumor-supplying vessels, stimulatory cytokines (e.g., interferon α and β), and immune checkpoint inhibition (e.g., PD-1 inhibitors) have shown clinical activity in many different types of cancer.

Several pathways influence the intensity of an immune reaction to prevent autoimmune reactions. Inhibitory pathways induce downregulation of T cell activation or effector functions (79). T cells with receptors recognizing non-self structures on tumor cells are the key players to trigger anti-tumor immune responses. Binding of the T cell receptor accompanied by a co-stimulatory signal leads to T cell activation. The tight control of this process is essential to inhibit excessive activation leading to autoimmune reactions, whereby the proteins CTLA-4 and PD-1 on T cells play major roles as brakes of T cell activation. Blocking CTLA-4 and/or PD-1 by antibodies can restore immune activation, referred to as immune checkpoint therapy (80, 81). Examples for antibodies that target PD-1 are Pembrolizumab or Nivolumab, applied in several types of cancer including tumors of the skin, kidney, bladder, head and neck, lung, and Hodgkin lymphoma (82).

Challenges of current immunotherapies are systemic autoimmune reactions, low response rates, tremendous costs, and application to patients with compromised immune systems. Loading immunotherapeutics onto nanoparticulate transporters can increase their therapeutic potential (83). Thus, currently nanoparticles are being investigated as transporters for antigens, adjuvants, or siRNA to activate the immune system (5). To target nanoparticles to PD-L1 expressing cancer cells, PD-1 antibody was not only used on nanoparticles as targeting ligand but also for disturbing the interaction between PD-L1 on tumor cells and immune cells (84).

Tumor accumulation of nanoparticulate immunotherapeutics can further be increased by targeting T cells in the circulation since leukocytes are the first cells intravenously applied nanoparticles get in contact with. Additionally, lymphocytes can deeply penetrate into the tumor tissue. Thus, nanoparticles targeting PD-1 expressed on T cells and inhibition of TGF-β signaling have been shown to increase survival of tumor bearing mice. With this approach dosing can be significantly reduced, thus limiting potential toxicity (85). In this context first pilot experiments have been performed to load T cells *ex vivo* with SPIONs as transporters for (immune modulatory) drugs to subsequently inject and guide them to the tumor area using an external magnetic field (86).

### Combination of Nanocarriers (SPIONs) With Hyperthermia and Radiotherapy

Mild hyperthermia can elicit cell death by denaturation of proteins and/or damage of DNA and other mechanisms, resulting in apoptosis (87). Inefficient blood flow and supply with oxygen through the quickly generated blood vessels in tumors results in an acidotic and nutrient-deprived milieu making cancer cells more thermo sensitive to acute increases in temperature than healthy cells (88). Major problem with conventional methods to induce hyperthermia is the generation of homogenous therapeutic temperatures deep in the tumor. Here, SPIONs can act as controllable heat source: in alternating magnetic fields, the magnetic polarity rapidly flips. However, there is some hysteretic loss involved in the flipping, revealing as heat. Thus, a tumor can be heated in alternating magnetic fields if preloaded with SPIONs. Although there are some reports on use of magnetic hyperthermia alone to treat and/or cure cancer in animal models, magnetic hyperthermia is often used in combination. Radiotherapy and hyperthermia have complementary effects: Poorly perfused tumor cores are sensitive to hyperthermia but resistant to ionizing radiation which depends on the formation of toxic oxygen radicals in well perfused areas. Also, in the S phase of the cell cycle tumor cells exhibit radioresistancy, but are highly sensitive to heat. Thus, hyperthermia can act as radiosensitizer to radioresistant cancer cells (89).

Radiosensitizers, such as histone deacetylase inhibitors, which inhibit DNA double strand repair can enhance the response of tumor cells to radiation through the prolongation of γ-H2AX foci as shown with polymer nanoparticles (90). Also, binding of radionuclids to SPIONs, particularly β emitters, induced DNA damage due to free radicals, resulting in apoptosis of target cells (91). Also, SPIONs have shown their potential as X ray-enhancer for low-dose irradiation therapy. After radiation the amount of toxic ROS in tumor cells with engulfed nanoparticles has substantially increased (92, 93).

## Summary

For efficient cancer treatment including long-term immune reactions, the immunogenicity of the tumor must be increased and the tolerance of the immune system against tumor associated antigens abrogated. Importantly, at the same time, immune compatibility has to be preserved. With nanoparticles as platform technology immunotherapeutics and/or chemotherapeutic drugs can be targeted towards the tumor. Compared to systemic application, the intratumoral drug concentration can be increased and healthy tissues spared from the drug related side effects by nanoparticle-mediated transportation (Figure 1B). Concurrent radiation and/or hyperthermia of the tumor induces cell death and increases immunogenicity of the tumor cells. Employing SPIONs as drug transporters enables multimodal therapy concepts since compounds of various therapeutic classes (e.g., chemotherapeutics, immune modulators, phototoxic compounds) can be bound and adapted to the individual profile of the patient. Using SPIONs as nanoparticle platform additionally enables monitoring of tumor targeting in MRI (Theranostics) (62, 63).

## Author Contributions

All authors listed have made a substantial, direct and intellectual contribution to the work, and approved it for publication.

### Conflict of Interest Statement

The authors declare that the research was conducted in the absence of any commercial or financial relationships that could be construed as a potential conflict of interest. The handling editor declared a shared affiliation, though no other collaboration, with the authors.
